# Pharmacogenomics-based subtype decoded implications for risk stratification and immunotherapy in pancreatic adenocarcinoma

**DOI:** 10.1186/s10020-024-01049-6

**Published:** 2025-02-19

**Authors:** Xing Zhou, Yuhao Ba, Nuo Xu, Hui Xu, Yuyuan Zhang, Long Liu, Siyuan Weng, Shutong Liu, Zhe Xing, Shuang Chen, Peng Luo, Libo Wang, Xinwei Han

**Affiliations:** 1 Department of Interventional Radiology, The First Affiliated Hospital of Zhengzhou University, Zhengzhou, Henan China; 2 Department of Pediatric Surgery, The First Affiliated Hospital of Zhengzhou University, Zhengzhou, China; 3 Center for Reproductive Medicine, The First Affiliated Hospital of Zhengzhou University, Zhengzhou, China; 4 Department of Hepatobiliary and Pancreatic Surgery, The First Affiliated Hospital of Zhengzhou University, Zhengzhou, Henan China; 5 School of Basic Medical Sciences, College of Medicine, Zhengzhou University, Zhengzhou, China; 6 Department of Neurosurgery, The Fifth Affiliated Hospital of Zhengzhou University, Zhengzhou, China; 7 Department of Oncology, Zhujiang Hospital, Southern Medical University, Guangzhou, China; 8 Department of Pancreatic Cancer, Tianjin Medical University Cancer Institute and Hospital, National Clinical Research Center for Cancer, Key Laboratory of Cancer Prevention and Therapy, Tianjin, China; 9 Interventional Institute of Zhengzhou University, Zhengzhou, Henan China; 10 Interventional Treatment and Clinical Research Center of Henan Province, Zhengzhou, Henan China

**Keywords:** Pancreatic adenocarcinoma, Pharmacogenomics, Immunotherapy, Molecular subtype, Precision medicine

## Abstract

**Background:**

With fatal malignant peculiarities and poor survival rate, outcomes of pancreatic adenocarcinoma (PAAD) were frustrated by non-response and even resistance to therapy due to heterogeneity across clinical patients. Nevertheless, pharmacogenomics has been developed for individualized-treatment and still maintains obscure in PAAD.

**Methods:**

A total of 964 samples from 10 independent multi-center cohorts were enrolled in our study. With drug response data from the profiling of relative inhibition simultaneously in mixtures (PRISM) and genomics of drug sensitivity in cancer (GDSC) databases, we established and validated multidimensionally three pharmacogenomics-classified subtypes using non-negative matrix factorization (NMF) and nearest template prediction (NTP) algorithms, separately. The heterogenous biological characteristics and precision medicine strategies among subtypes were further investigated.

**Results:**

Three pharmacogenomics-classified subtypes after stable and reproducible validation, distinguished in six aspects of prognosis, biological peculiarities, immune landscapes, genomic variations, immunotherapy and individualized management strategies. Subtype 2 was close to immunocompetent phenotype and projected to immunotherapy; Subtype 3 held most favorable outcomes and metabolic pathways distinctively, promising to be treated with first-line agents. Subtype 1 with worst prognosis, was anticipated to chromosome instability (CIN) phenotype and resistant to chemotherapeutic agents. In addition, ITGB6 contributed to subtype 1 resistance to 5-fluorouracil, and knockdown of ITGB6 enhanced sensitivity to 5-fluorouracil in in vitro experiments. Ultimately, appropriate clinical stratified treatments were assigned to corresponding subtypes according to pharmacogenomic transcripts. Some limitations were not taken into account, thus needs to be supported by more research.

**Conclusion:**

A span-new molecular subtype exploited for PAAD uncovered an insight into precise medication on ground of pharmacogenomics, and highly refined multiple clinical management strategies for specific patients.

**Supplementary Information:**

The online version contains supplementary material available at 10.1186/s10020-024-01049-6.

## Introduction

With intensifying obesity and aging, the incidence of pancreatic adenocarcinoma (PAAD) is increasing, but its treatment prognosis has not improved significantly, with a 5-year survival rate of only 11%, which is the 7th cancer-related deaths in worldwide, and even expected to become the second leading burden of cancer mortality by 2030 in western countries(Siegel et al. 2021; Park et al. 2021; Rahib et al. 2014). Limited to the insidious onset and lack of ideal screening tools, the vast majority of patients have already locally advanced or metastasized at the time of diagnosis, and 10-15% of patients with resectable or borderline resectable disease have a 5-year survival of only 20% after surgery(Park et al. 2021; Mizrahi et al. 2020). The emergence of the PARP inhibitor olaparib has shed light on the treatment of PAAD, and results from phase 3 clinical trial have shown that it significantly prolongs median progression-free survival in patients with *BRCA*-mutated metastatic PAAD (7.4 months vs. 3.8 months)(Golan et al. 2019; Zhu et al. 2020). Moreover, attribute to its complex stroma and highly heterogeneous tumor microenvironment (TME), inappropriate treatment caused a 40% incidence of grade 3 or higher adverse drug events, and we urgently need to screen sensitive patients for individualized treatment(Golan et al. 2019). With the popularization of next-generation sequencing, some molecular subtypes of PAAD have been proposed, but their clinical application is still very restricted(Chan-Seng-Yue et al. 2020; Collisson et al. 2019). Therefore, it is urgent need to deepen the understanding of molecular heterogeneity and then develop new stratified management and individualized treatment strategies to observably improve the clinical outcome of PAAD.

With generalization of the FOLFIRINOX strategy (consists of oxaliplatin, irinotecan, fluorouracil, and leucovorin) and gemcitabine plus nab-paclitaxel as the first-line regimen in patients with advanced PAAD, appropriate patients have verified significantly prolonged survival(Park et al. 2021; Mizrahi et al. 2020; Tempero et al. 2021). Recently, numerous studies have been devoted to exploring whether those patients are more suitable for FOLFIRINOX or gemcitabine plus nab-paclitaxel, but no consensus has been reached(Shi et al. 2020; Klein-Brill et al. 2022). In this setting, pharmacogenomics harbors the expect to precision medication with consideration of genetic determinants and guides stratified management by reducing molecular heterogeneity within subgroups(Roden et al. 2019; Hassouni et al. 2019). Additionally, chemoresistance associated with heterogeneity across genomics and TME appears a growing issue, and even first-line drug gemcitabine may develop resistance within the initial weeks during chemotherapy(Ireland et al. 2016; Binenbaum et al. 2015; Assaraf et al. 2019; Seebacher et al. 2021). Integrating pharmacogenomics, Ding RB et al. established four nasopharyngeal carcinoma subtypes with distinct molecular characteristics, drug responsiveness, and radiosensitivity, and validated potential subtype-specific treatment options through patient-derived organoids to further serve precision medicine(Ding et al. 2021). Recently, Gu Z et al. conducted a high-throughput drug screening through 2,248 drug response data from 56 cell models of head and neck squamous cell carcinoma patients-derived and 18 immortalized cell lines, and then developed and verified biomarkers that could predict gene-drug correlations, drug sensitivity, and drug resistance(Gu et al. 2022). Our previous study also revealed the attractive prospect of pharmacogenomics in individualized precision medicine strategies directed towards lung adenocarcinoma subtypes. However, pharmacogenomics has not received sufficient attention in the stratified management and individualized medication of PAAD(Ge et al. 2022).

In this study, based on the drug susceptibility data of 104 PAAD cell lines for 8 classical PAAD drugs from the profiling of relative inhibition simultaneously in mixtures (PRISM) and genomics of drug sensitivity in cancer (GDSC) drug response datasets, we obtained 46 drug response-featured genes via differential analysis between response and resistance groups. Using non-negative matrix factorization (NMF) algorithm, we identified three novel PAAD subtypes and further investigated their biological difference in prognosis, functional characteristics, immune landscape, genomic variation, treatment response and individualized management strategy. And small-scale in vitro experiments to further validated. This revealed the molecular heterogeneity of individuals with diverse drug sensitivities from a broader perspective, and highly refining stratification management and precision medication strategies for PAAD patients in clinical practice.

## Materials and methods

### Data source and processing

With consideration of establishing model with sufficient samples, processed expression data and corresponding clinical information from gene expression omnibus (GEO, https://www.ncbi.nlm.nih.gov/geo/) were downloaded and integrated into the GEO-Meta cohort with batch correction, including GSE21501 (*n* = 102), GSE28735 (*n* = 42), GSE57495 (*n* = 63), GSE71729 (*n* = 125), GSE78229 (*n* = 49), GSE79668 (*n* = 51) and GSE85916 (*n* = 79). Expression files in FPKM format for TCGA-PAAD (*n* = 176) were downloaded from the UCSC Xena website (https://xenabrowser.net/datapages/) and converted to log2 (TPM + 1) format. The corresponding clinical and raw mutation files were also obtained from UCSC Xena, and copy number alterations (CNAs) data were obtained from the FireBrowse website (www.firebrowse.org). Processed validation cohort consisted of PACA-AU (*n* = 82) and PACA-CA (*n* = 195) from International Cancer Genome Consortium (ICGC, https://dcc.icgc.org/) got rid of batch effect after normalization, and then merged into ICGC-Meta cohort. The detailed explanation for nine datasets used in this study was displayed in Table S1.

Following National comprehensive cancer network (NCCN) and Chinese society of clinical oncology (CSCO) guideline recommendations, we selected 17 classical drugs for PAAD and further acquired drug response data of PAAD cell lines from the profiling of relative inhibition simultaneously in mixtures (PRISM) and genomics of drug sensitivity in cancer (GDSC) websites(Corsello et al. 2020; Yang et al. 2013). Inspired by RECIST 1.1 system(Schwartz et al. 2016), which classifies the chemotherapeutic response in solid tumors into complete response, partial response, and stable disease/progression disease, we stratified the cell lines into sensitive, partial response, and resistant groups according to the half maximal inhibitory concentration (IC50), log10 (IC50), concentration for 50% of maximal effect (EC50), and log10 (EC50) of the candidate drugs with reference to previous experience(Ye et al. 2021). Where the sensitive group was defined as owning IC50, log10 (IC50), EC50, and log10 (EC50) of the cell line below the mean − 0.5 standard deviation (SD) of the drug, the resistant group was identified as possessing sensitivity of the cell line exceeded the mean + 0.5 SD of the drug, and the partial response group was between mean + 0.5 SD and mean − 0.5 SD of corresponding drug.

### Identification of drug response-featured genes

Next, we downloaded cancer cell lines (CCLs) expression data within PRISM from Cancer Cell Line Encyclopedia (CCLE, https://sites.broadinstitute.org/ccle/datasets) database and further transformed them into log2 (TPM + 1). Expression data for PAAD cell lines in the GDSC database were obtained from GDSC1000 resource and normalized by robust multi-array average (RMA) algorithm. Based on differential expression analysis of sensitive and resistant PAAD cell lines for the 17 candidate drugs, we filtered consistently up-regulated and consistently down-regulated genes in the PRISM and GDSC databases using thresholds of *P* < 0.05 and |log2fold change (FC)| > 0.5 and named them as drug response-featured genes for subsequent studies after de-duplication.

### Clustering subtypes based on drug response-featured genes

Based on the expression files of drug response-featured genes in the GEO-Meta cohort with the largest sample, the non-negative matrix factorization (NMF) was employed to perform consensus clustering and feature extraction using NMF package. Referring to a previous study, we set the initial parameters to rank = 2–7, iteration = 100, and then selected the optimum cluster number according to the cophenetic coefficients(Brunet et al. 2004). In addition, silhouette width was used to evaluate the validity of the clustering results, and samples with negative values were removed(Rousseeuw and Silhouettes 1987).

### Weighted correlation network analysis (WGCNA)

To search for featured genes of different subtypes, we constructed a scale-free network of expression matrix using the WGCNA package(Langfelder and Horvath 2008). After raising the intergenic correlation matrix to the soft threshold power, we further established the adjacency matrix. Following, we calculated topological overlap matrix (TOM) by the TOMsimilarity algorithm and clustered genes with the same co-expression pattern into corresponding color modules by dissimilarity (1-TOM). In addition, we merged similar modules using cutreeDynamic algorithm and further picked the most relevant modules for each drug response-featured subtype via correlation analysis for the next study.

### Validation by nearest template prediction (NTP) algorithm

To obtain stable characteristics gene for the three subtypes, combined with differential analysis, we retained the intersected genes between subtype-specific module and differentially up-regulated gene as signature gene. Further, based on the signature genes described above, we used the NTP algorithm to generate our drug response-featured subtypes in the TCGA-PAAD and ICGC-Meta cohorts to verify the robustness and reproducibility of our classification system(Hoshida 2010). Kaplan-Meier survival curves were used to evaluate the accuracy of the predicted subtypes in the validation cohorts. In addition, Subclass Mapping (Submap) analysis on Gene pattern (https://www.genepattern.org) was conducted to verify consistency inside predictive subtypes(Hoshida et al. 2007), and the proportion of subtype samples were calculated to distinguish the compatible distribution of three subtypes in validation sets.

### Functional enrichment analysis

To explore the functional characteristics of each subtype, we performed gene set variation analysis (GSVA) and gene set enrichment analysis (GSEA) for the featured genes of each subtype based on the C2-C8 gene sets in the Molecular Signatures Database (MSigDB, https://www.gsea-msigdb.org/gsea/msigdb). After that, we selected the most significant pathways for each subtype according to the log2FC of difference analysis in GSVA pathway and the Normalized Enrichment Score (NES) of the GSEA pathway to visualize.

### Immune landscape of different subtypes

Using markers provided by Charoentong P et al.(Charoentong et al. 2017), we assessed the infiltration abundance of 28 immune cells between different subtypes via single sample gene set enrichment analysis (ssGSEA). Furthermore, we also performed seven common immune assessment tools including TIMER, CIBERSORT, CIBERSORT_ABS, quanTIseq, MCPcounter, xCell, and EPIC to validate ssGSEA results. Then, we recruited several critical immune indicators for antitumor immunity and immunotherapy from previous studies(Ott et al. 2019; Thorsson et al. 2018), including antigen presentation score (APS), T-cell inflammatory signature (TIS), T cell and B cell receptor diversity (TCR.Richness and BCR.Richness), as well as co-stimulators and co-inhibitors expression. In addition, immune-cancer cycle (CIC) was implemented based on research by Karasaki T et al. to simulate immune response to tumor in vivo for subtypes(Karasaki et al. 2017).

### Assessment of immunotherapeutic efficacy

To evaluate the potential response to immunotherapy in different subtypes, we additionally collected six immunotherapy cohorts such as GSE100797, GSE115821, GSE126044, GSE136961, GSE140901, and GSE35640. Applying Submap algorithm, we speculate on the efficacy of immunotherapy by comparing the similarity of expression matrices between the drug response-featured subtypes and different immunotherapeutic response populations.

### Genomic alteration analysis

Based on the multi-omics profiles of TCGA-PAAD cohort, we sequentially explored the tumor mutation burden (TMB), the distribution of high-frequency mutational signature 1 (age), mutational signature 2 (APOBEC), mutational signature 3 (BRCA1/2 mutations) and mutational signature 6 (DNA MMR deficiency) in PAAD(Alexandrov et al. 2013), the mutation frequency of top 15 high-frequency mutation genes, copy number amplification and deletion fragments with copy number alterations (CNAs) frequencies greater than 20%, and some classical genes located in these high-frequency CNAs fragments. In addition, several global CNAs indicators including fraction of genome alteration (FGA), fraction of genome gained (FGG), and fraction of genome lost (FGL) as well as copy number gain and loss burden at the chromosomal arm and focal levels were further investigated.

### Drugs sensitivity analysis

In order to develop potential drugs for specific subtype, we integrated three databases including Genomics of Drug Sensitivity in Cancer (GDSC1 and GDSC2) and PRISM, as well as three cohorts, TCGA, GEO-Meta and ICGC-Meta. The ridge regression algorithm implemented in the oncoPredict package was used for predicting drug response(Maeser et al. 2021). This predictive model was trained on transcriptional expression profiles and drug response data of cancer cell lines with a satisfied predictive accuracy were evaluated by default 10-fold cross-validation, and then estimated of clinical drug response using the expression data of three cohorts. Meanwhile, correlation analysis between the IC50 of drugs and the expression of subtype-specific signature genes was implemented to filter out the gene most significantly associated with drug resistance.

### Cell culture and reagents

Human PAAD cell lines BxPC-3 and AsPC-1 were obtained from Pricella Biotechnology Co., Ltd. (Wuhan, China) and cultured in RPMI-1640 with 10% FBS contained. All cell lines were cultured in the indicated humidified environment (37 °C, 5% CO2). 5-fluorouracil was purchased from MCE company and was freshly diluted in DMSO to a 50 mM stock.

### Transient transfection of siRNA

To silence ITGB6 in cancer cells, ITGB6 specific siRNA (si-ITGB6) and control siRNA were transfected into BxPC-3 and AsPC-1 cells. Lipofectamine 3000 (Invitrogen; Thermo Fisher Scientific, L3000–015) was utilized as a transfection carrier. The transfection efficiency was confirmed via RT-PCR analysis.

### RT‒qPCR

Following the manufacturer’s instructions, total RNA was isolated with RNAiso Plus reagent (Takala, Dalian, China). Then, RNA was reverse transcribed to cDNA with PrimeScript RT Master Mix Kit (Takala, Dalian, China) and subsequently subjected to RT-qPCR using GoTaq qPCR Master Mix (Vazyme, Nanjing, China) according to the manufacturer’s instructions. The primer sequence of ITGB6 was provided: Forward: TCTCCTGCGTGAGACACAAAGG; Reverse: GAGCACTCCATCTTCAGAGACG. All data were analyzed and normalized to GAPDH.

### CCK-8 assay

The viability of cancer cells was measured with a CCK-8 kit according to the manufacturer’s instructions. Cancer cells were seeded in 96-well plates with 2 × 10^4^ /well. After pretreatment with different concentrations of 5-fluorouracil in BxPC-3(0、10、20、40、80、160、320)µM and AsPC-1(0、1、5、10、20、40、80)µM for 24 h, 10 µl CCK-8 solution was added to each well. Cell viability was examined after treatment with 80µM (BxPC-3) or 20µM (AsPC-1) 5-fluorouracil in 0, 24, 28 ,72 and 96 h in each treatment group. All assays were performed in quintuplicate. Cell viability was examined at 450 nm using a microplate reader.

### Clinical delicacy management

So far, researchers have constructed numerous prognostic models based on a specific biological pathway such as autophagy and glycosylation to serve precision medicine(Zhang et al. 2021; Jonckheere and Seuningen 2018). However, limiting by the strong heterogeneity of PAAD and the generalizability of the models, these models have not been applied to clinical practice. Therefore, to achieve stratified management and individualized treatment, based on our drug response-featured subtypes, we collected 86 published prognostic models of PAAD and searched for the best-performing prognostic biomarkers in each subtype by the mean C-index of the GEO-Meta training cohort as well as the TCGA-PAAD and ICGC-Meta validation cohorts.

### Statistical analysis

All data collection, normalization, statistical analysis and plotting tasks were left to R 4.2.1 software. Pearson correlation was conducted to evaluate the relationship between two continuous variables. Kruskal-Wallis test, Wilcoxon rank-sum test or student’s t test were implemented to figure out the statistical differences between subtypes. All *P* (two-sided) < 0.05 in this study were considered statistically significant.

## Result

### Identification of drug response-featured genes

**Fig. 1:**
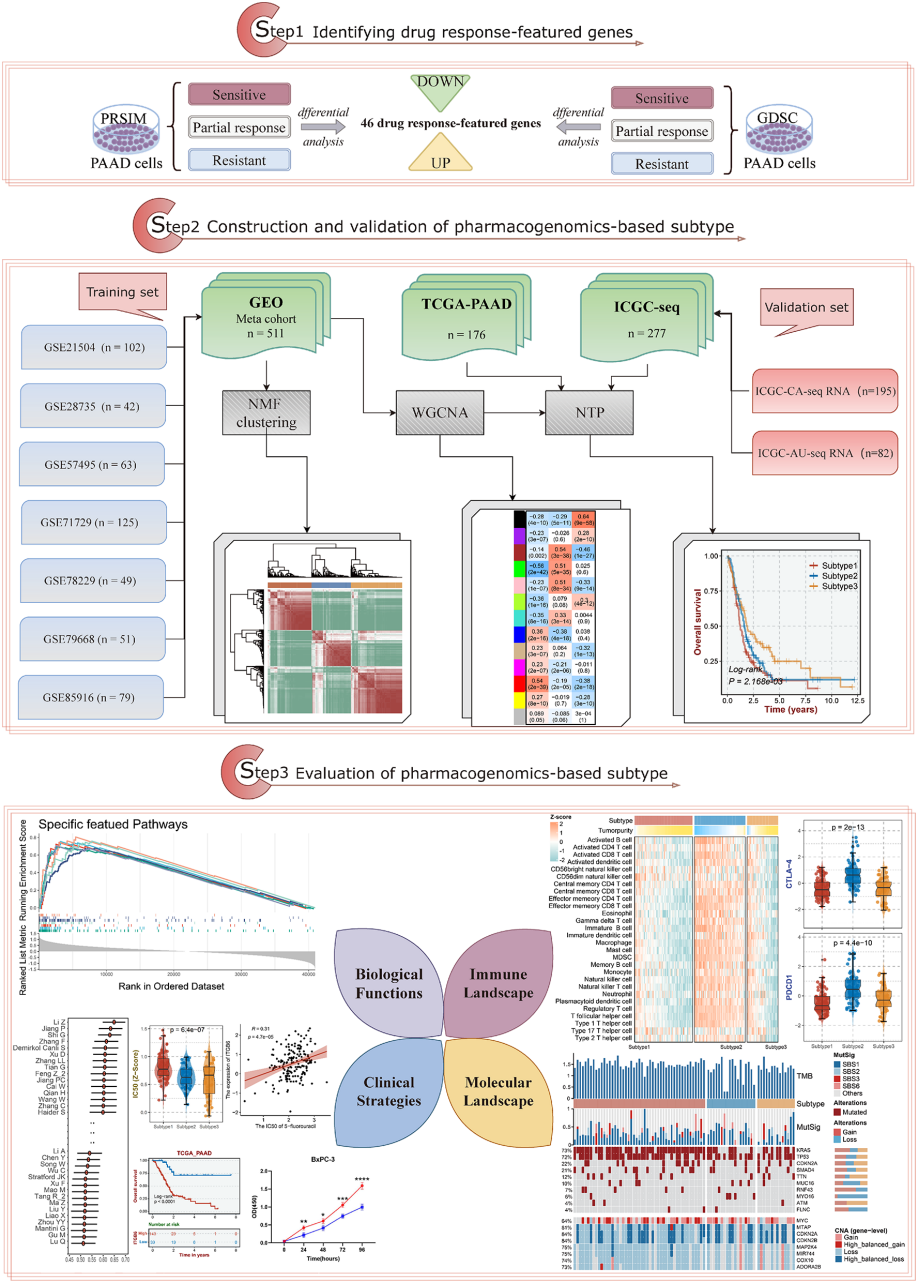
Workflow of the current study. To start with, we initially made use of the drug response of cell lines from PRISM and GSDC to generate a total of 46 drug response-featured genes by respectively applying differential analysis to sensitive, partial response and resistant group. Next, with three PAAD cohorts procured from GEO, TCGA and ICGC platforms severally, a molecular subtype based on pharmacogenomics was constructed with cohort of GEO-Meta data by NMF algorithm. Then to validate the reproducibility of the subtype, two cohorts including TCGA-PAAD and ICGC-Meta cohort combined of ICGC-CA and ICGC-AU data were utilized via WGCNA and then NTP analysis. Ultimately, sets of four dimensions analysis, exclusive of biological functions, immune landscape, molecular landscape, and clinical management strategies were implemented to present the full insight into pharmacogenomics-classified subtype

According to workflow shown in Fig. 1, sensitive, partial response, and resistant cell lines for 17 candidate drugs from guidelines were spotted depending on PRISM and GDSC datasets. As a result, 5-fluorouracil, cisplatin, epirubicin, gemcitabine, irinotecan, olaparib, oxaliplatin, a total of seven classical chemicals held differential expression genes with consistency of up- or down-regulation between sensitive and resistant groups (Table S2). Subsequently, they were utilized to generate 46 drug response-featured genes.

### Three subtypes established on drug response-featured genes

**Fig. 2:**
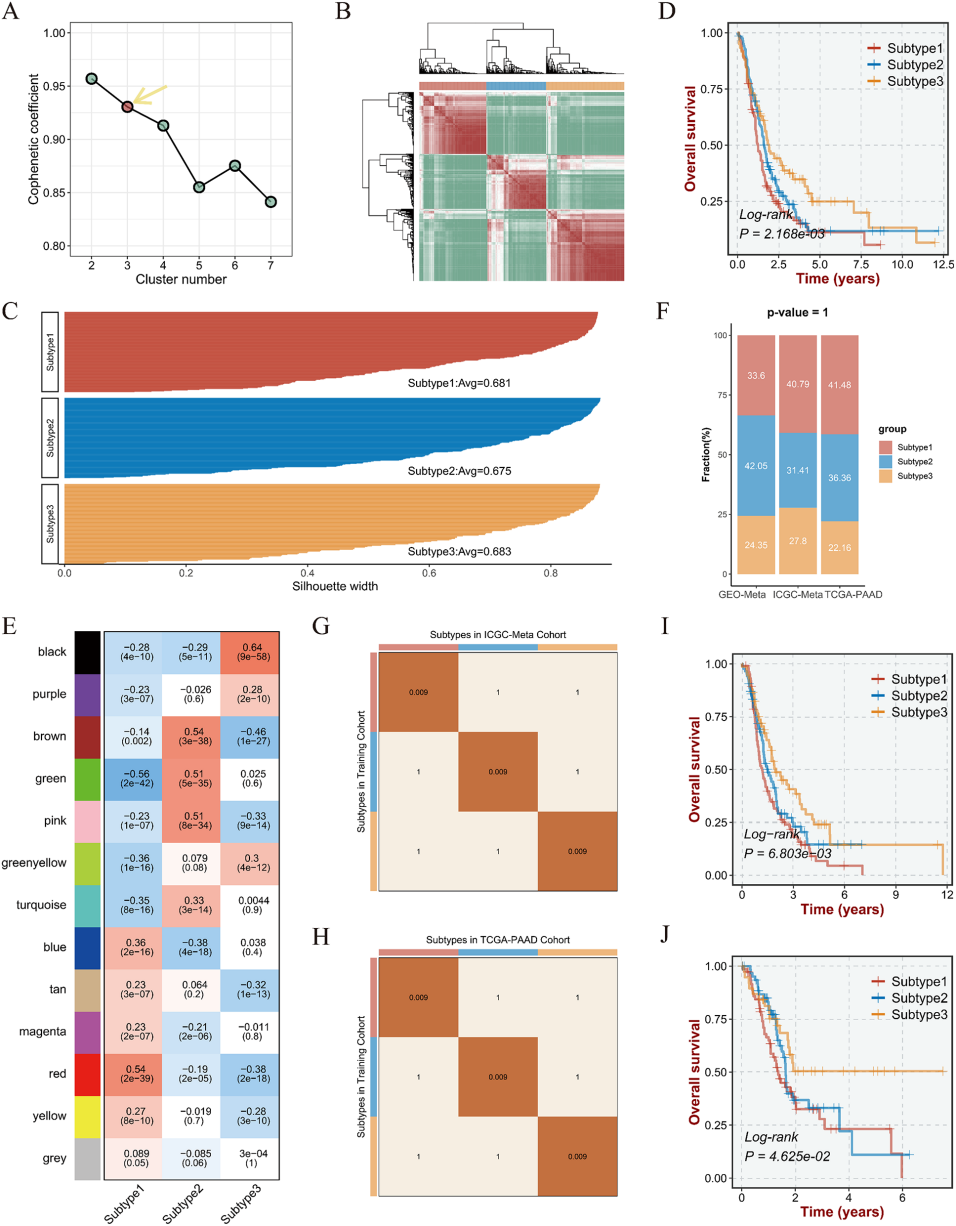
Identification of drug response-featured genes and construction of pharmacogenomics-classified subtype. **A.** Among cluster number ranging k = 2–7 with cophenetic coefficients calculating by NMF algorithm, the yellow arrow regarded k = 3 as the optimal cluster number. **B.** The consensus map revealed that samples were clustered into corresponding subtypes, as they all scored higher in exact subtypes. **C.** The specific modules were picked for each subtype according to the most absolute correlations, and the scatterplots of genes in specific modules for each subtype were plotted to show the high degree of correlation. **D.** Kaplan-Meier (K-M) survival curve of overall survival for three subtypes depending on GEO-Meta cohort inspected by log-rank test. **E.** WGCNA analysis provided correlation coefficient as well as *P* value for each module inside three subtypes. **F.** The column chart illustrated the distribution of proportion of three subtypes in total samples of each cohort. **G-H.** The Submap illustrated similarities of transcriptional pattern between training set and ICGC-Meta (the upper) or TCGA-PAAD (the bottom) cohort, and the rigorous correlations can be verified by more significant *P* value (*P* < 0.01, correction method: Bonferroni). **I-J.** The K-M survival curves of ICGC-Meta (the upper) and TCGA-PAAD cohort (the bottom) by NTP algorithm for validation

With 46 drug response-featured genes input calculating by NMF algorithm, k = 3 was identified as the optimal cluster number of clusters (Fig. 2A). The consensus map (Fig. 2B) presented strong consistency intra-subtype and difference inter-subtype, thus interpreting the basis of genomic hierarchy in PAAD patients. Also, Fig. 2C showed silhouette widths of Subtype 1, 2 and 3 maintained at a high level of 0.68, 0.67, and 0.68 respectively, implicating great innate consistency inside. The survival curve (Fig. 2D) demonstrated that three subtypes held distinctive life expectancy, therein Subtype 3 featuring the most favorable prognosis, Subtype 1 with the poorest, and Subtype 2 with the intermediate.

### To verify repeatability and reliability of subtype in two cohorts

Initially, WGCNA algorithm was implemented to identify module-trait genes for subtypes based on pharmacogenomics (Fig. 2E). Considering that higher coefficient related to more rigorous pertinency, then modules with coefficient greater than 0.5 were picked for each subtype, such as red module to Subtype 1, brown, green and pink three modules to Subtype 2, and black module to Subtype 3. The scatter plots (Supplementary Fig. 1A-E) illustrated the correlation between corresponding module-trait genes and subtypes. Integrated with the subtype-specific up-regulated genes obtained by differential analysis, we obtained 228, 668, and 284 signature genes for Subtypes 1, 2 and 3, respectively (Table S3). Based on the predictive subtypes from TCGA-PAAD and ICGC-Meta cohorts by NTP, module-trait genes spectacularly enriched corresponding subtypes (Supplementary Fig. 1F-G). In addition, the distribution of subtypes in two validation cohorts unfolded identical to training set (Fig. 2F). Submap analysis demonstrated the similarity of corresponding subtypes between GEO-Meta cohort for training and two validation cohorts (Fig. 2G-H). And their prognosis was also revealed optimal consistency by Kaplan-Meier survival curve (Fig. 2I-J). This allows us to believe that we have captured the best fitting subtypes from the GEO-Meta cohort in the TCGA-PAAD and ICGC-Meta cohorts.

**Fig. 3:**
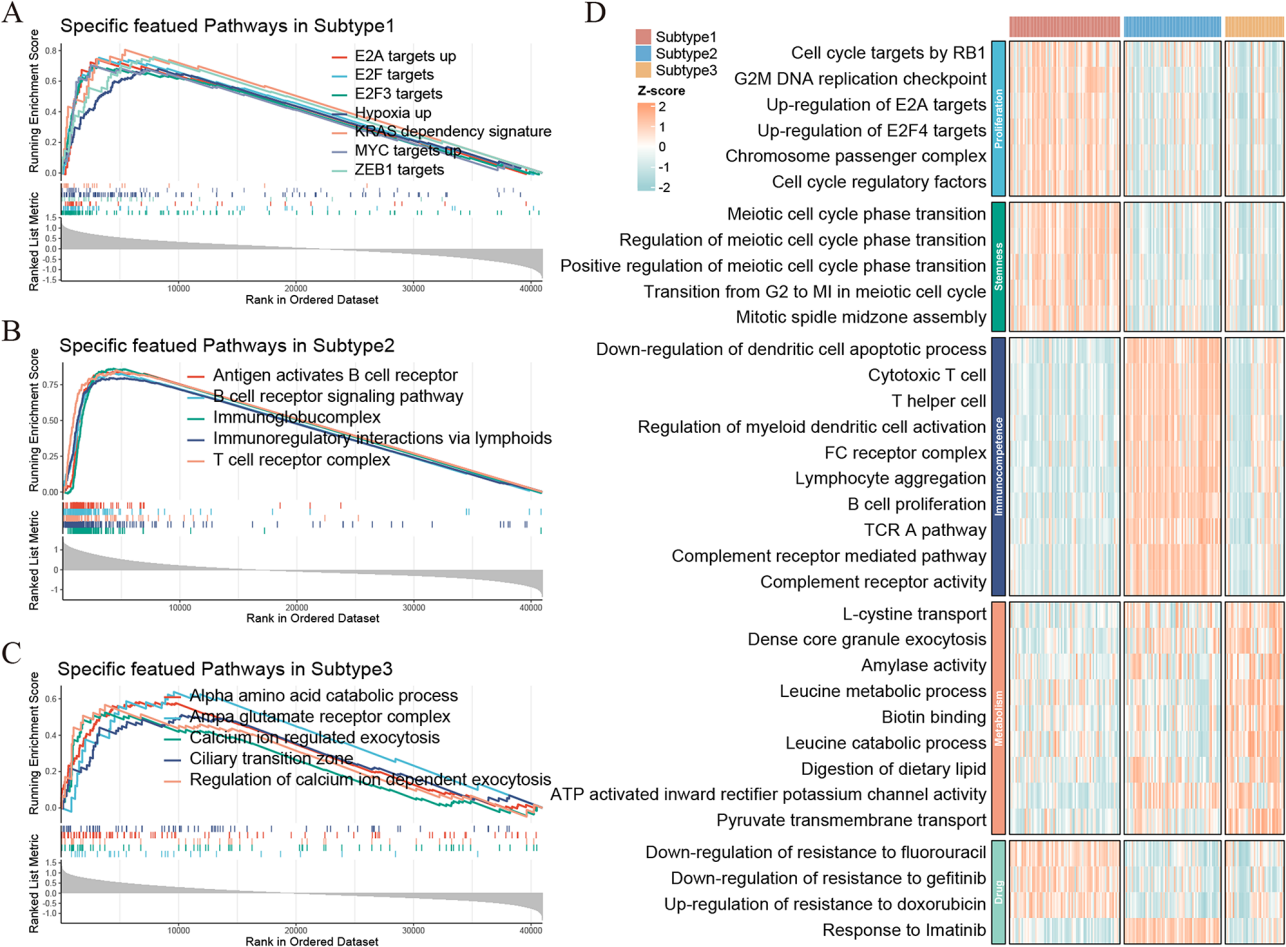
Biological function of three subtypes. **A-C.** GSEA plots with smooth upward curves demonstrated up-regulated pathways in three subtypes respectively. **D.** Pathways heatmap deciphered biological functions was specifically gathered in three subtypes, including proliferation, stemness, immunocompetence, metabolism and drug

### Specific biological functions of subtype relative to others

Involving total of 32,847 gene sets on MSigDB, we investigated specific biological functions on pharmacogenomics-classified subtypes via GSEA algorithm. According to GSEA plot (Fig. 3A), Subtype 1 manifested the proliferative pathways associated with E2F, E2A and E2F3 targets by cell cycle regulating promoters and so on. Subtype 2 enriched on immunocompetent pathways, to name a few, B cell receptor signaling, immunocomplex and T cell receptor complex were included (Fig. 3B). Furthermore, it found that the metabolic pathways related to alpha amino acid catabolic process, calcium ion regulated exocytosis, ciliary transition zone scored the highest NES in Subtype 3 (Fig. 3C). The above results were also confirmed by GSVA analysis, Subtype 1 accumulated dense z-score in proliferation and stemness, Subtype 2 was defined as immunocompetence as well as Subtype 3 as metabolism (Fig. 3D).

### Multiple immune landscapes

**Fig. 4:**
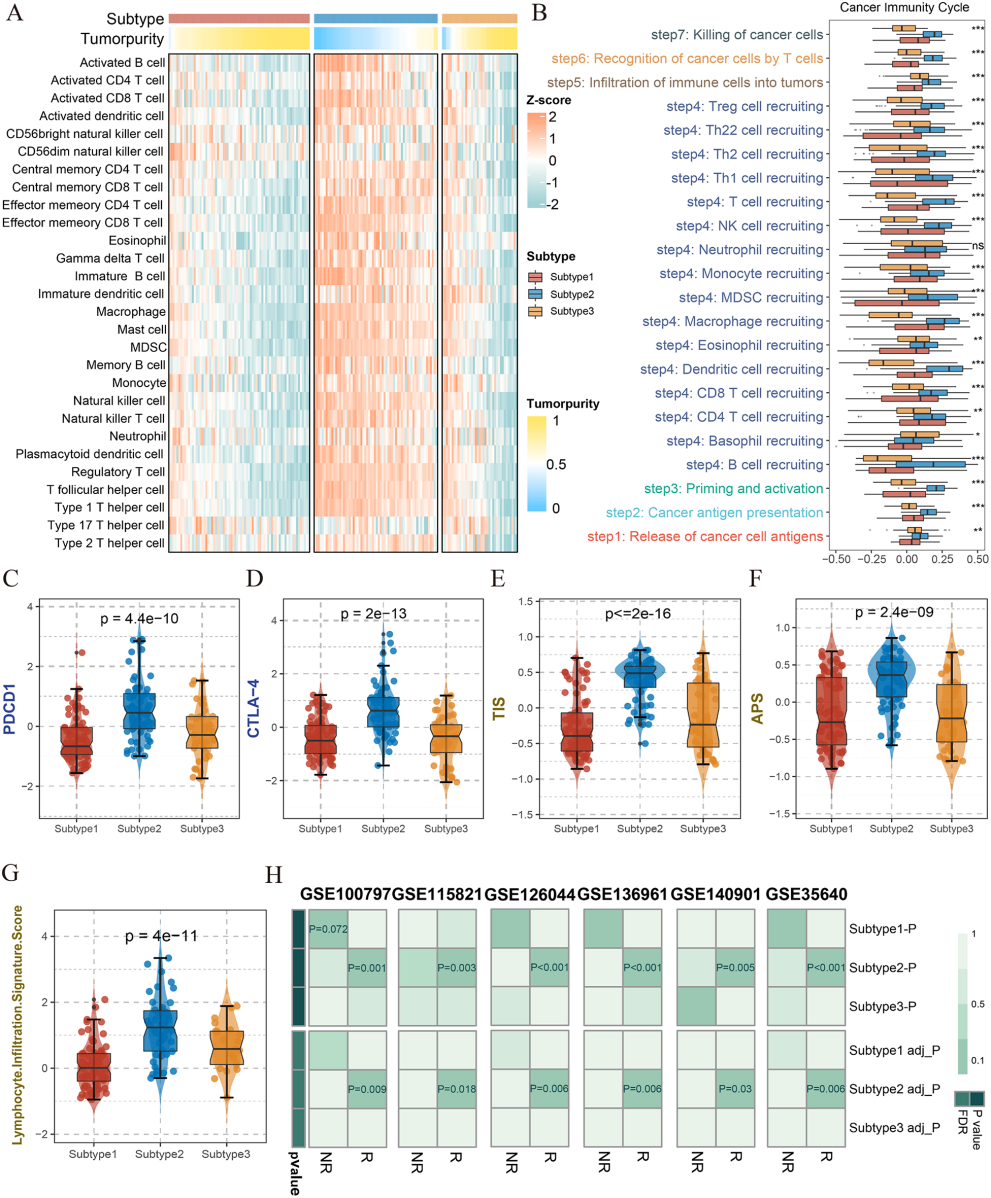
Immune landscape of subtype based on pharmacogenomics. **A.** The gram by ssGSEA algorithm revealed 28 immune cells infiltration abundance in three subtypes. Obviously, Subtype 1 and 3 appeared deficient in immune abundance infiltration, while Subtype 2 was endowed with considerable immune cells in TME. **B.** Immunogram of CIC bare out the same results from ssGSEA analysis, that extreme activity of immune system to tumors in Subtype 2, intermediate activity in Subtype 3, and even non-response in Subtype 1. **C-D.** The boxplots illustrated the distribution of three subtypes in PDCD1 and CTLA-4, respectively. **E-G.** The boxplots illustrated the distribution of three subtypes in TIS, APS, and lymphocytic infiltration score, respectively. **H.** The heatmap illustrated simulated immunotherapeutic efficacy of three subtypes, the one enjoyed the significant P value (*p* < 0.05), including normal version and Bonferroni corrected, may effectively respond to immunotherapy in reality

Now that bioinformatics delineated an immune and inflammatory function with Subtype 2, it was essential to investigate the comprehensive immune landscapes of three subtypes. The analysis by ssGSEA displayed Subtype 2 was spotlighted with the striking infiltration of almost all immune cells, but obviously apart from T helper 17 cell (Fig. 4A). Intriguingly, its tumor purity of most samples remained lowest among three subtypes, showing consistency with immune cell abundance in sync. As for Subtype 1 and 3, they were embowed with low-density of immune cell infiltration as well as highly dense tumor purity. The boxplot also illustrated that both innate and adaptive immune cells infiltrated richly in Subtype 2, followed by Subtype 3, and least by Subtype 1 (Supplementary Fig. 1H). Likewise, TIMER, CIBERSORT, CIBERSORT_ABS, quanTIseq, MCPcounter, xCell and EPIC yielded consistent infiltration tendency with ssGSEA algorithm (Supplementary Fig. 2A).

The CIC theory by Karasaki et. condensed the anti-tumor immunity to 7 steps, and Subtype 2 exhibited distinctively higher activity in each step (Fig. 4B). Several canonical immune checkpoint molecules such as PD-L1, PDCD1, CTLA-4, LAG3, MHC and co-stimulatory molecules expressed extensively in Subtype 2 (Fig. 4C-D, Supplementary Fig. 2B-D, G). In a comprehensive perspective of the reference indicators for immunotherapy recruited from Thorsson and his collegues(Thorsson et al. 2018), TIS, APS, TCR.Shannon, BCR.Shannon, and Lymphocytic Infiltration Scores were significantly higher in Subtype 2, suggesting patients represented by Subtype 2 may response better to immunotherapy (Fig. 4E-G, Supplementary Fig. 2E-F).

To confirm the hypothesis, we enrolled six extension cohorts including GSE100797, GSE115821, GSE126004, GSE136961, GSE140901 and GSE35640. As a result, their Submap analysis demonstrated Subtype 2 was endowed with transcriptional similarity to immunotherapy responsive population, suggesting more effective immunotherapy (Fig. 4H).

### Molecular characterization of three subtypes

**Fig. 5:**
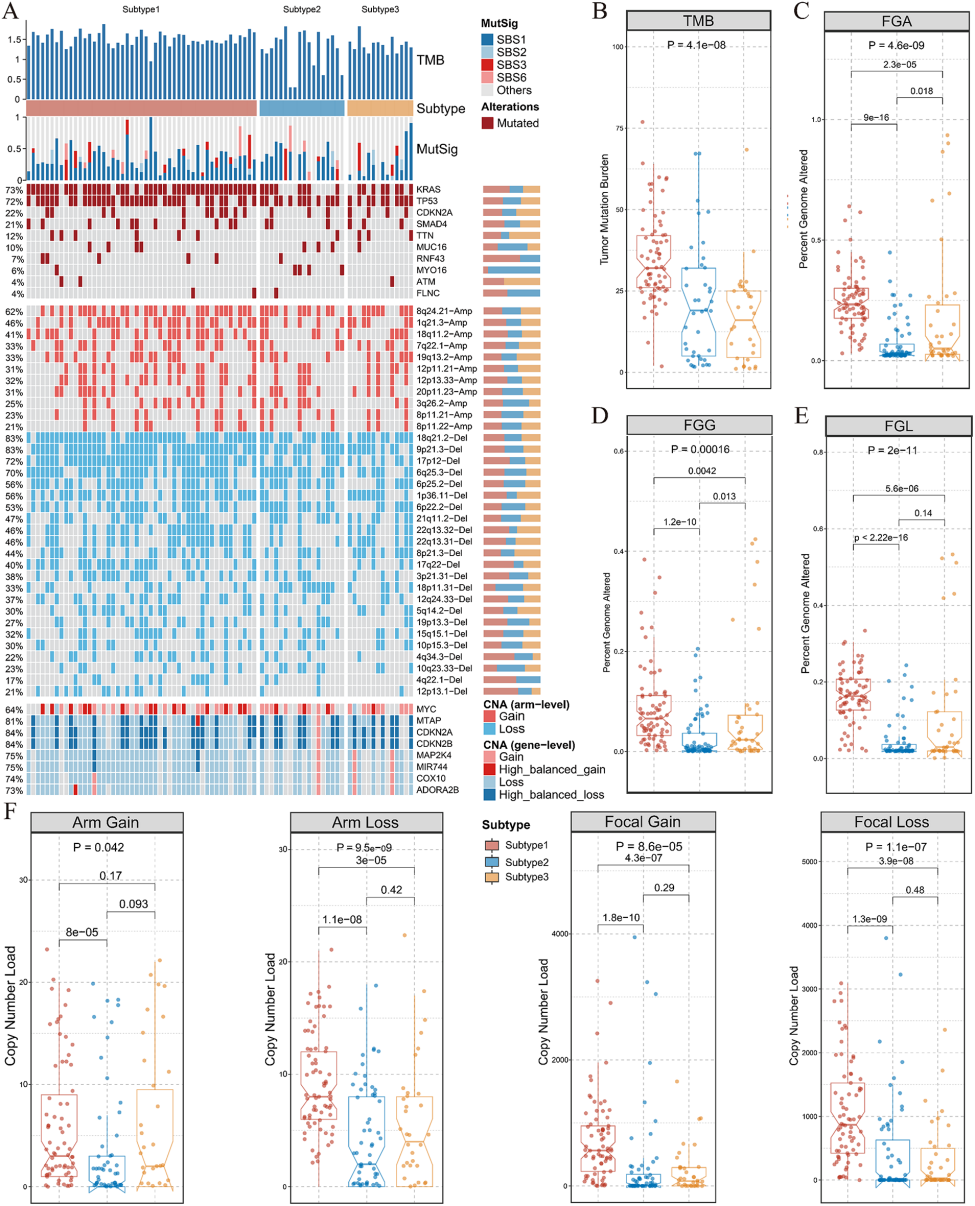
Molecular landscape of pharmacogenomics-classified subtype. **A.** An integrative molecular and mutation landscape of three subtypes, consisted of TMB bar plots, mutation signature proportion plots, waterfall plots of mutation genes and amplification as well as deletion on chromosome arms, and essential CNAs in three subtypes. **B.** TMB across three subtypes in the form of boxplot. **C-E.** The difference of FGA, FGG, and FGL in subtype based on pharmacogenomics. **F.** Genomic alterations at focal and broad level, including focal gain, focal loss, arm gain, and arm loss

Next, we characterized multi-omics landscapes with sets of genomic mutation and CNAs sequentially. Figure 5A exhibited the comprehensive CNAs of pharmacogenomics-based subtypes, and four canonical mutations: KRAS, TP53, CDKN2A and SMAD4 appeared evenly across three subtypes. Based on the boxplots showing fraction of TMB, genome altered (FGA), fraction of genome gained (FGG), fraction of genome loss (FGL), Subtype 1 stood out that its mutation accounted for the largest proportion of genome-wide (Fig. 5B-E). Likewise, alterations at focal and arm level elaborated the same result (Fig. 5F), so Subtype 1 was characterized with high chromosome instability (CIN), poorest prognosis, and proliferative and stem-cell-like features. What’s more, Subtype 1 bore the most frequency of mutational signature 1 (APOBEC), and it was reported that both CIN and APOBEC were highly associated with adverse outcomes in cancers(Drews et al. 2022; Lindskrog et al. 2021) (Fig. 5A). Thus, the worst outcome, high CIN and malignance could be identified as specific to Subtype 1.

### Drug sensitivity for subtype 1 and 3

**Fig. 6:**
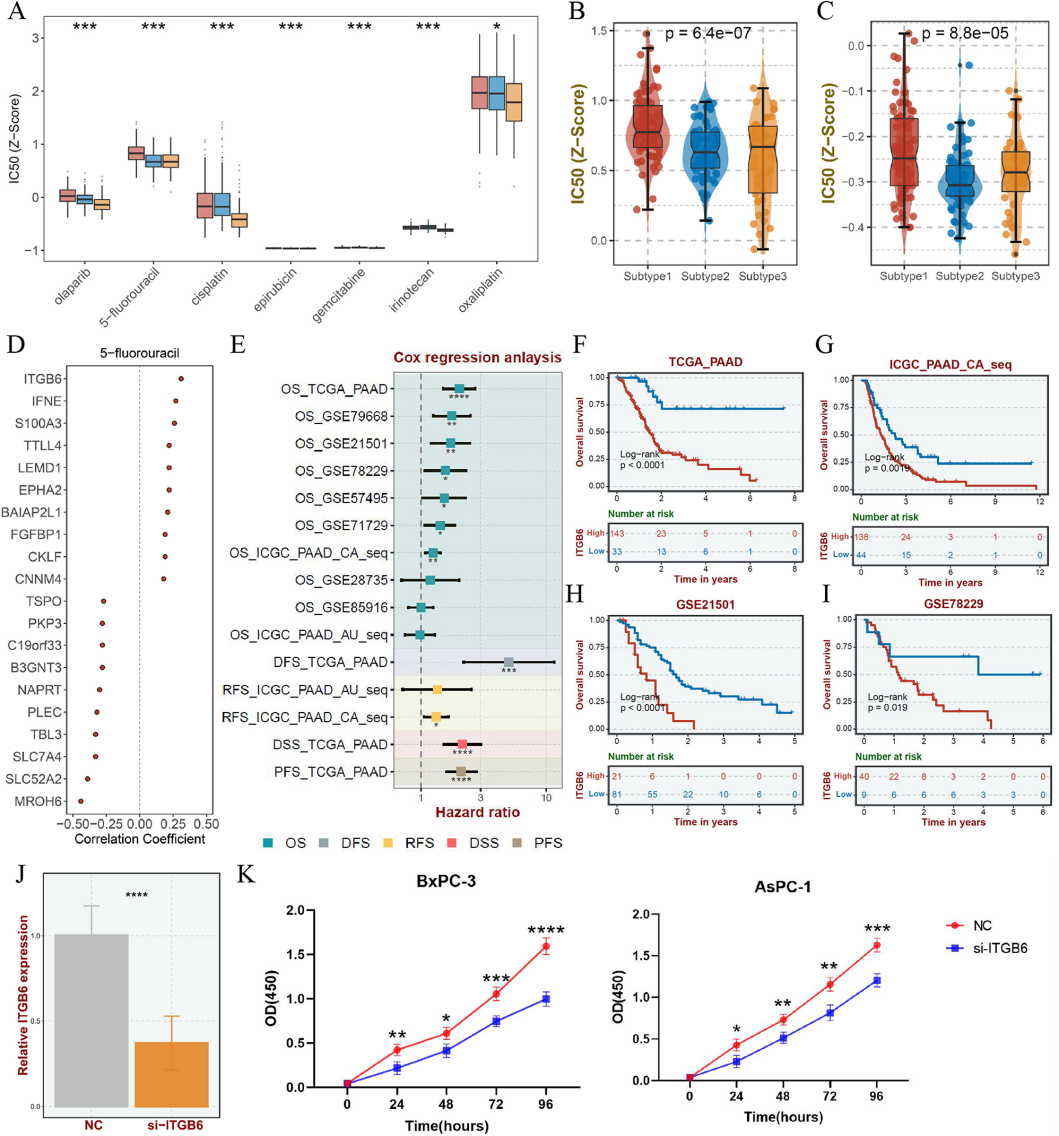
Drug sensitivity for pharmacogenomics-classified Subtype 1 and 3. **A.** The difference of IC50 value of olaparib, 5-fluorouracil, cisplatin, epirubicin, gemcitabine, irinotecan and oxaliplatin in subtype based on pharmacogenomics. **B-C.** The IC50 value of 5-fluorouracil in PRISM or GDSC across three subtypes in TCGA in the form of boxplot. **D.** The top 20 signature genes in Subtype 1 with high correlation with the IC50 value for 5-fluorouracil. **E.** The Cox analysis for ITGB6 in multiple PAAD datasets. **F-I.** K-M survival curves of overall survival for ITGB6 inspected by log-rank test. **J.** mRNA expression level of ITGB6 in knockdown group (si-ITGB6) and control group (si-NC). **K.** Absorbance at 450 nm wavelength after CCK8 treatment in different treatment groups at different time

Afterwards, we developed potential and sensitive agents for Subtype 1 and 3, which showed non-response and even resistance to immunotherapy. The multiple chemicals exhibited low IC50 in Subtype 3, suggesting it might bear more responsive to routine agents of PAAD (Fig. 6A). However, consistent with stemness biological functions as well as general CIN phenotype in Subtype 1, multiple canonical chemicals may be nonsensitive to Subtype 1, especially 5-fluorouracil possessing high IC50 value in five of six cohorts (Fig. 6B-C, Supplementary Fig. 3A-D). In order to shed light on the potential mechanisms of drug resistance, we conducted the correlation analysis between IC50 of 5-fluorouracil and signature genes of Subtype 1 displayed ITGB6 was positive correlation with IC50 value implying its potential effect to facilitate drug resistance (Fig. 6D, Supplementary Fig. 3E-F). Besides, the Cox and K-M survival analysis also demonstrated that high expression of ITGB6 held unfavorable prognosis (Fig. 6E-I, Supplementary Fig. 3G-Q). We then carried out small-scale in vitro experiments to further validate above result, and then the results of CCK-8 assay presented good agreement with our informatics-based results. Specifically, ITGB6 silencing by siRNA transfection could promote sensitivity to 5-fluorouracil in BxPC-3 and AsPC-1 cells (Fig. 6J-K, Supplementary Fig. 3R-S). In brief, it suggested that for PAAD patients, we are promising to clinically implement an individualized treatment based on our pharmacogenomics-classified subtype.

**Fig. 7:**
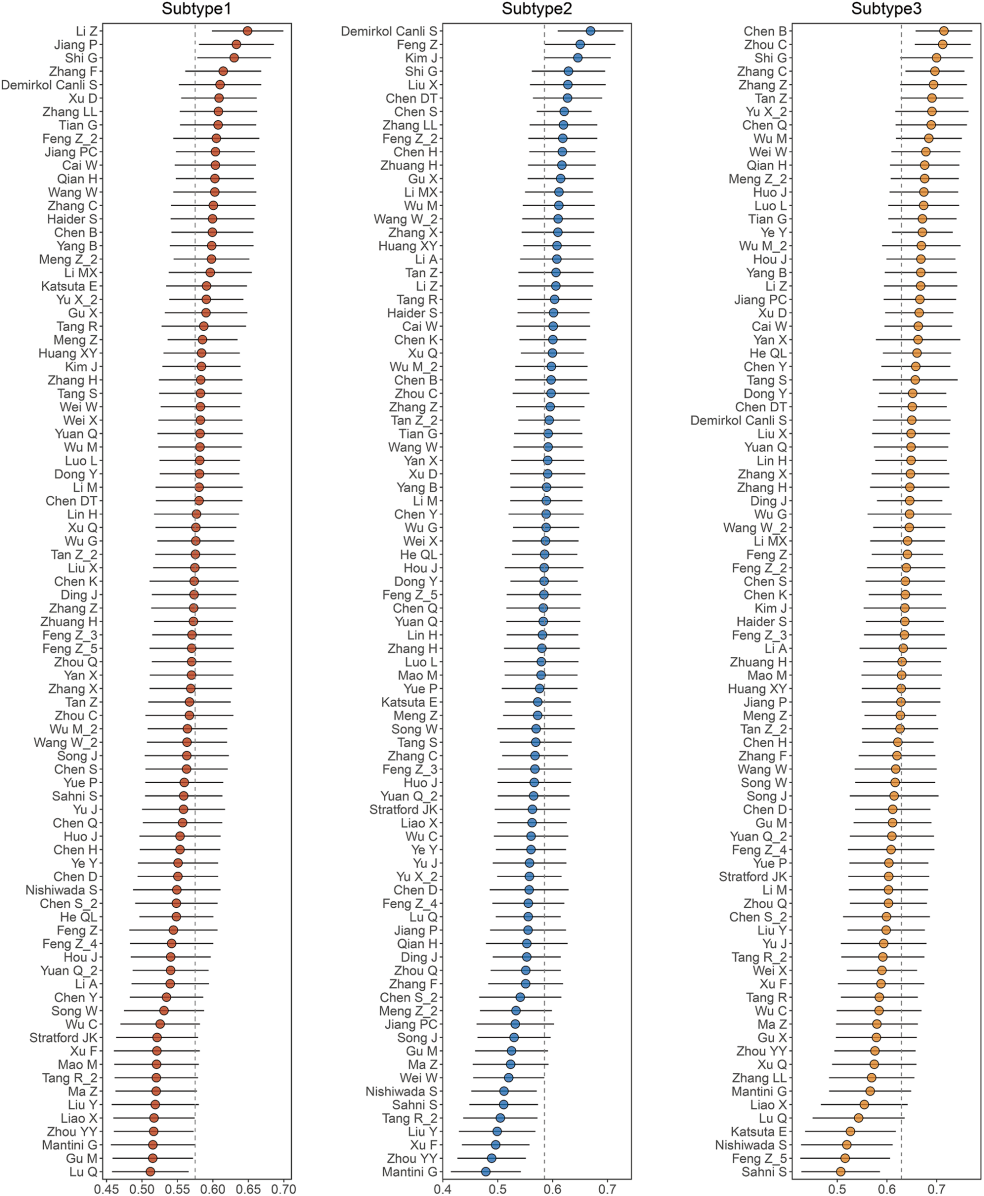
Clinical management strategies for subtype based on pharmacogenomics. Exhibition of multiple signatures for PAAD in three subtypes, the higher C-index scored in one subtype, the more compatible for application to clinical delicacy management for the certain subtype

### Clinical delicacy management

Owing to prevalent application of bioinformatics technologies and development of PAAD sequencing datasets, amounts of prognostic models of PAAD have been established currently for stratified management and precision medicine(Li et al. 2021; Demirkol Canli et al. 2020; Chen et al. 2021). Depending on 86 PAAD transcription-trait signatures collected by our team(Wang et al. 2022), more compatible and matchable clinical delicacy management were searched by mean C-index in training set and two validation sets. To begin with, five-mRNA risk prognostic signature by Li Z bore highest C-index for Subtype 1 (Fig. 7), and also responsible for predicting T lymphocyte infiltrations(Li et al. 2021). Subtype 2 matched the 20-gene prognostic score developed by Demirkol Canli S which can assist to quantitate survival status and guide clinical strategies(Demirkol Canli et al. 2020) (Fig. 7). And an optimal insight into Subtype 3 was revealed by Chen B (Fig. 7), establishing two signatures based on differentially expressed immune-related genes (DEIRGs) for prognostic prediction(Chen et al. 2021).

## Discussion

Despite oncology medication and treatment technology have been improved for decades, the cancer-related fatality of pancreatic adenocarcinoma incredibly progressed more than double from 1990 to 2017, along with doubling global incidence and prevalence(Collaborators 2019; Klein 2021). Still, current first-line therapeutic agents have greatly prolonged the life and quality of patients with advanced PAAD, but severe issues including chemoresistance and adverse drug reactions across genomics and TME were not resolved yet(Golan et al. 2019; Ireland et al. 2016; Assaraf et al. 2019). Therefore, an optimal stratification regimen holds the expects to assign propriate patients for corresponding treatments based on pharmacogenomic heterogeneity. In this study, we classified three pharmacogenomics-based subtypes: (1) Subtype 1, identified as CIN phenotype, scored higher genomic alterations, along with the poorest prognostic profile of short survival and malignant biological peculiarities; (2) Subtype 2 was endowed with immunocompetent phenotype and promisingly responded under immunotherapy; (3) Subtype 3 held the most favorable prognosis and potential efficacy with consensus drugs in clinical guidelines. Finally, the relatively appropriate clinical management strategies were proposed for each subtype as heterogeneity across the three, which helped see through specialties intra-subtype.

Owing much to next generation sequencing technology and accessibility of drug susceptibility data, pharmacogenomics was paid attentions in the field of oncological hierarchical management(Ding et al. 2021; Gu et al. 2022; Ge et al. 2022). Based on drug respond data from PRISM and GDSC, we first classified immortalized PAAD cell lines as sensitive, partially response and resistant groups depending on response to 17 candidate drugs. After applying differential analysis between sensitive and resistant groups for each drug, we procured a total of 46 drug response-featured genes with consistent up- and down-regulations. Based on drug response-featured genes, three pharmacogenomics-classified subtypes were generated by NMF algorithm in the largest GEO-Meta cohort.

Verification of robustness of molecular subtype demands prompt solution in machine-learning world, while some published researches are subject to single cohort data and cannot be extended to clinical practice(Topham et al. 2021). Given this, after implementing WGCNA analysis along with differential expression analysis for identification of subtype-specific module-trait genes, both TCGA-PAAD and ICGC-Meta cohorts were brought into NTP algorithm for verifying reproductivity and stability of pharmacogenomics-based subtype. Thus, not only did the transcriptional characteristic of multi-center cohorts maintain analogical, but the subtype proportions and clinical traits were also consistent with training set.

Depending on 32,847 gene sets from MSigDB, we can have an insight into the heterogenous biological functions across three subtypes via GSVA and GSEA analysis. Subtype 1 might enrich in proliferation and stemness pathways, suggesting malignance tendency. Meanwhile, Subtype 2 primarily enriched in immunocompetent pathways and Subtype 3 gathered on the metabolism pathways. Considering that multiple mesenchymal components of PAAD shape the heterogeneous TME and mediate genesis of pharmacoresistance(Ireland et al. 2016; Seebacher et al. 2021), it was essential to discern the immune landscape across three subtypes. According to ssGSEA analysis, Subtype 2 was expected presumably as immunocompetent phenotype with variable infiltration of immune cell, and then other seven common algorithms were applied and validated immune abundance in Subtype 2. As active antigen presentation deducted from expression of MHC molecules and APS, and then BCR.Shannon, TCR.Shannon as well as TIS showed widely distribution of immune effector cells in Subtype 2. Meanwhile, CIC provided an overall process of immunocidal program and confirmed the immunoactivity of Subtype 2 anew(Charoentong et al. 2017; Ott et al. 2019; Thorsson et al. 2018; Karasaki et al. 2017). Given the extensive expression of PDCD1, PD-L1, and CTAL-4, Subtype 2 was projected to align with immune checkpoint blockades (ICBs) therapy recommendation against the low response situation of the unscreened PAAD cohort currently(Morrison et al. 2018). On the ground of collected 6 cohorts with ICBs therapy, we predicted immunotherapeutic response of three subtypes by applying Submap analysis on Gene Pattern website. Of them, Subtype 2 delineated similar transcriptional pattern and effective response to immunotherapy in six cohorts with significant p-values (*p* < 0.05) after Bonferroni corrected rather than other subtypes. It implied that Subtype 2 may respond promisingly to ICBs represented by PD-1, and effect favorably on socioeconomic benefit.

Depending on multi-omics data for TCGA-PAAD cohort, the integrative mutational landscape of pharmacogenomics-classified subtype agreed with published researches that the most frequent mutated genes are KRAS, TP53, CDKN2A, and SMAD4, the four driven factors generally recognized. It was worth noting that only some samples in Subtype 1 appeared mutation of ATM, a tumor suppressor functioning in cell cycle checkpoint(Negrini et al. 2010). It was reported that carrying pathogenic variants of ATM increase risk of pancreatic cancer. Thus, our study of Subtype 1 indicated possible clinical traits and therapy for ATM variants individuals with PAAD. Notably, with analysis of FGA, FGG and FGL, Subtype 1 appeared highly approximate CIN characteristic, concordant with alterations at focal and arm levels. Coincidentally, Subtype 1 held the worst outcome and enriched in drug resistant pathways showing consistency with previous research which revealed that tumors of CIN associated with malignance, metastasis, therapy resistance(He et al. 2018; Sansregret et al. 2018; Bakhoum et al. 2018). Therefore, we identified Subtype 1 as CIN phenotype. At the focal and arm levels, we noticed that amplification occurred the most frequently at 8.q24.21, 1q21.3, 18q11.2, etc., while deletion frequently at 18q21.2, 9p21.3, 17p12, etc. Classical oncogenes such as MYC, MTAP, CDKN2A/B, and MAP2K4 were found on these frequently mutated segments of chromosomes, and mutation of CDKN2A exclusively appeared in Subtype 3. In summary, our study mapped an integral genome alteration in subtypes based on pharmacogenomics, and figured out CIN phenotype for Subtype 1.

Since Subtype 1 and 3 displayed non-response to ICBs, GDSC and PRISM datasets were applied to develop appropriate drugs in the aim of precise medicine. Notably, guideline agents were highly effective in Subtype 3, suggesting that it may be more compatible with standard chemotherapy. Subtype 1 exhibited resistance to routine chemotherapeutic agents and discovered the ITGB6 contributed resistance to 5-fluorouracil. Consistent with previous research which revealed that ITGB6 associated with migration, invasiveness and therapy resistance(Eijck et al. 2024; Lin et al. 2022). Most importantly, in vitro experiments with two PAAD cell lines (BxPC-3 and AsPC-1) also consistently illustrated that cell sensitivity to 5-fluorouracil was observably enhanced after knockdown of ITGB6, indicating that our precision treatment strategy for PAAD derived from pharmacogenomic-based subtypes has good prospects.

These days, with the development of high-throughput sequencing technique and the availability of public databases, numerous signatures on PAAD emerged with the goal of individualized management. Subtype 1 was promisingly concordant with the five-mRNA risk signature by Li Z which established on prognosis-related mRNA expression via the multivariate Cox regression analysis and also proposed two critical genes ANLN and MYEOV associated with T lymphocyte infiltrations(Li et al. 2021). Subsequently, Subtype 2 fitted a 20-gene prognostic score by Demirkol Canli S which indicated higher expressive score accompanied with lower abundance of immune cell infiltration, the similar situation with immune landscape of Subtype 2 (Demirkol Canli et al. 2020). And the signature by Chen D can predict survival status based on DEIRGs for Subtype 3 (Chen et al. 2021).

In this study, three subtypes with different prognostic based on pharmacogenomics were established on 46 drug response-featured genes. The three subtypes had wide heterogeneity in biological function, immune landscape, genomic variation, and treatment response. Based on this, we developed different stratified management and individualized treatment strategies for each of the three types of patients. However, there were certainly some shortcomings in this study. Although neoadjuvant chemotherapy and radiation therapy are widely used in the treatment of PAAD, the two strategies were not taken into consideration. Thus, in order to reduce the affect as far as possible we utilized multiple published signatures to refine clinical management. And drug response-featured genes were not integrally validated at the molecular level in this study. Pharmacogenomics-based subtypes needs to be supported by more research evidence.

## Conclusion

Establishing three reproducible and pharmacogenomics-stratified subtypes with stratified prognostic characteristics, each of them had different biological functions. Subtype 1 was characterized as CIN phenotype with enrichment in cell proliferation and stemness pathways, resistance to chemotherapy as well as poorest prognosis. Subtype 2 enriched in immunocompetent pathways and held potential immunotherapy implications, and Subtype 3 gathered in metabolic pathways with higher susceptibility to guideline agents. Ultimately, our subtypes also contributed to different therapeutic strategies for each subtype with the goal of personalized treatment and screening potential therapeutic agents for immunotherapy.

## Electronic supplementary material

Below is the link to the electronic supplementary material.

Supplementary Material 1

Supplementary Material 2

## Data Availability

Public data used in this work can be acquired from the from the UCSC-Xena (https://xenabrowser.net/datapages/), GEO (https://www.ncbi.nlm.nih.gov/geo/), ICGC (https://dcc.icgc.org/) and the FireBrowse database (www.firebrowse.org). Other data supporting the findings of this study are available from the corresponding author upon reasonable request.

## Abbreviations

PAAD: Pancreatic adenocarcinoma
PRISM: Profiling of relative inhibition simultaneously in mixtures
GDSC: Genomics of drug sensitivity in cancer
NMF: Non-negative matrix factorization
NTP: Nearest template prediction
CIN: Chromosome instability
TME: Tumor microenvironment
GEO: Gene expression omnibus
CNAs: Copy number alterations
ICGC: International Cancer Genome Consortium
NCCN: National comprehensive cancer network
CSCO: Chinese society of clinical oncology
IC50: Inhibitory concentration
EC50: Maximal effect
SD: Standard deviation
CCLs: Cancer cell lines
CCLE: Cancer Cell Line Encyclopedia
RMA: Robust multi-array average
WGCNA: Weighted correlation network analysis
TOM: Topological overlap matrix
Submap: Subclass Mapping
GSVA: Gene set variation analysis
GSEA: Gene set enrichment analysis
MSigDB: Molecular Signatures Database
NES: Normalized Enrichment Score
ssGSEA: Single sample gene set enrichment analysis
APS: Antigen presentation score
TIS: T-cell inflammatory signature
TMB: Tumor mutation burden
FGA: Fraction of genome alteration
FGG: Fraction of genome gained
FGL: Fraction of genome lost
MHC: Major histocompatibility complex
CIC: Cancer-immune cycle
