# Supplementary material for: Pharmacogenomics-based subtype decoded implications for risk stratification and immunotherapy in pancreatic adenocarcinoma
Source: Mol Med. 2025 Feb 19;31:62. doi: 10.1186/s10020-024-01049-6 (PMC11837470; doi:10.1186/s10020-024-01049-6)
Supplement: Supplementary file 2 — Supplementary Material 2 [file 10020_2024_1049_MOESM2_ESM.docx]

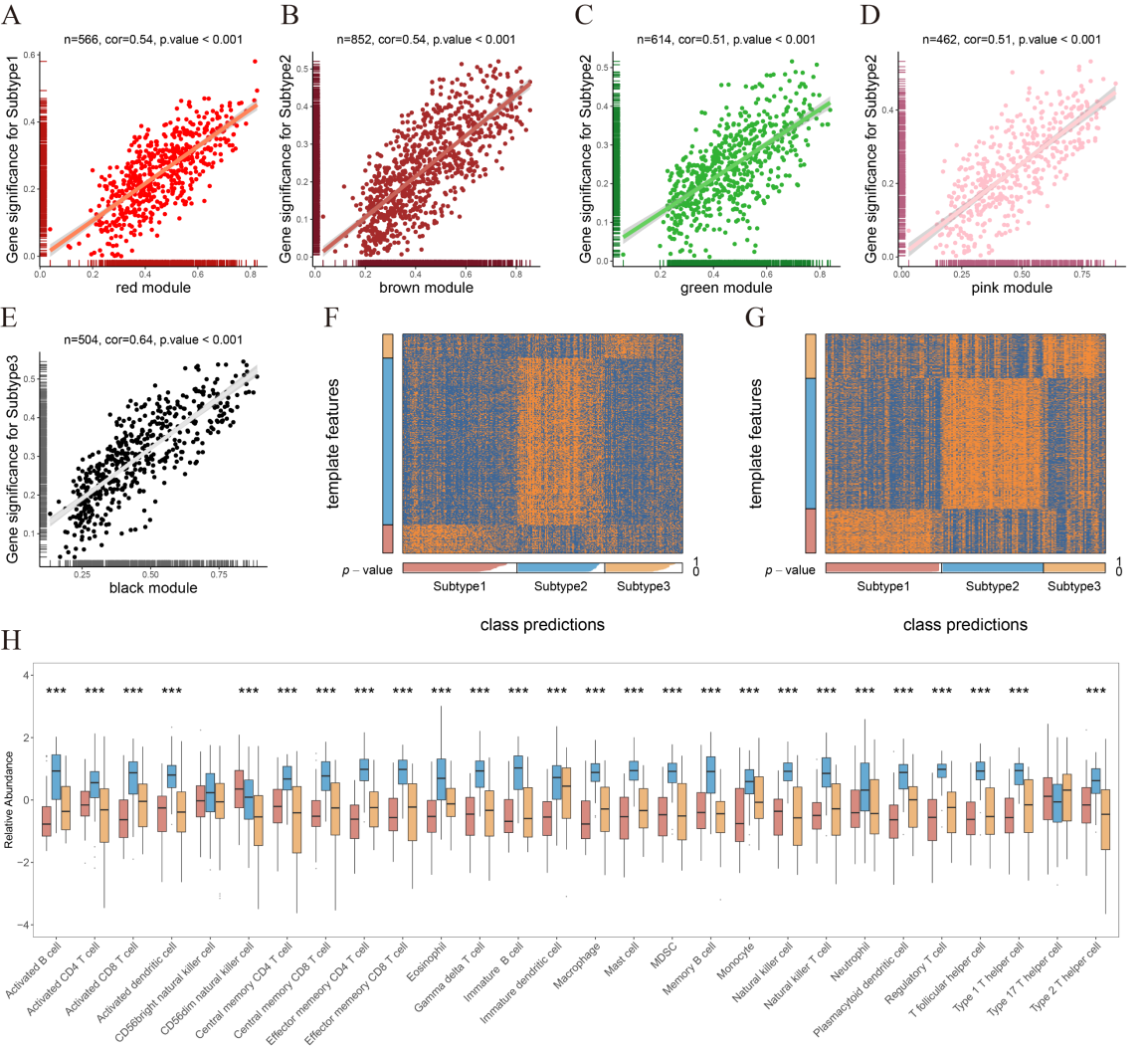


**Supplementary Fig. 1 A-E.** The scatter plots showed correlation of subtype-specific module genes, of which modules’ correlation coefficient higher than 0.5, and Subtype 1 corresponding to red module, Subtype 2 to black, brown and green, as well as Subtype 3 to pink module. **F-G.** The heatmaps illustrated subtype-specific module-trait genes expressing distribution across three subtypes. **H.** Immune cells infiltration across three subtypes in the form of boxplot.


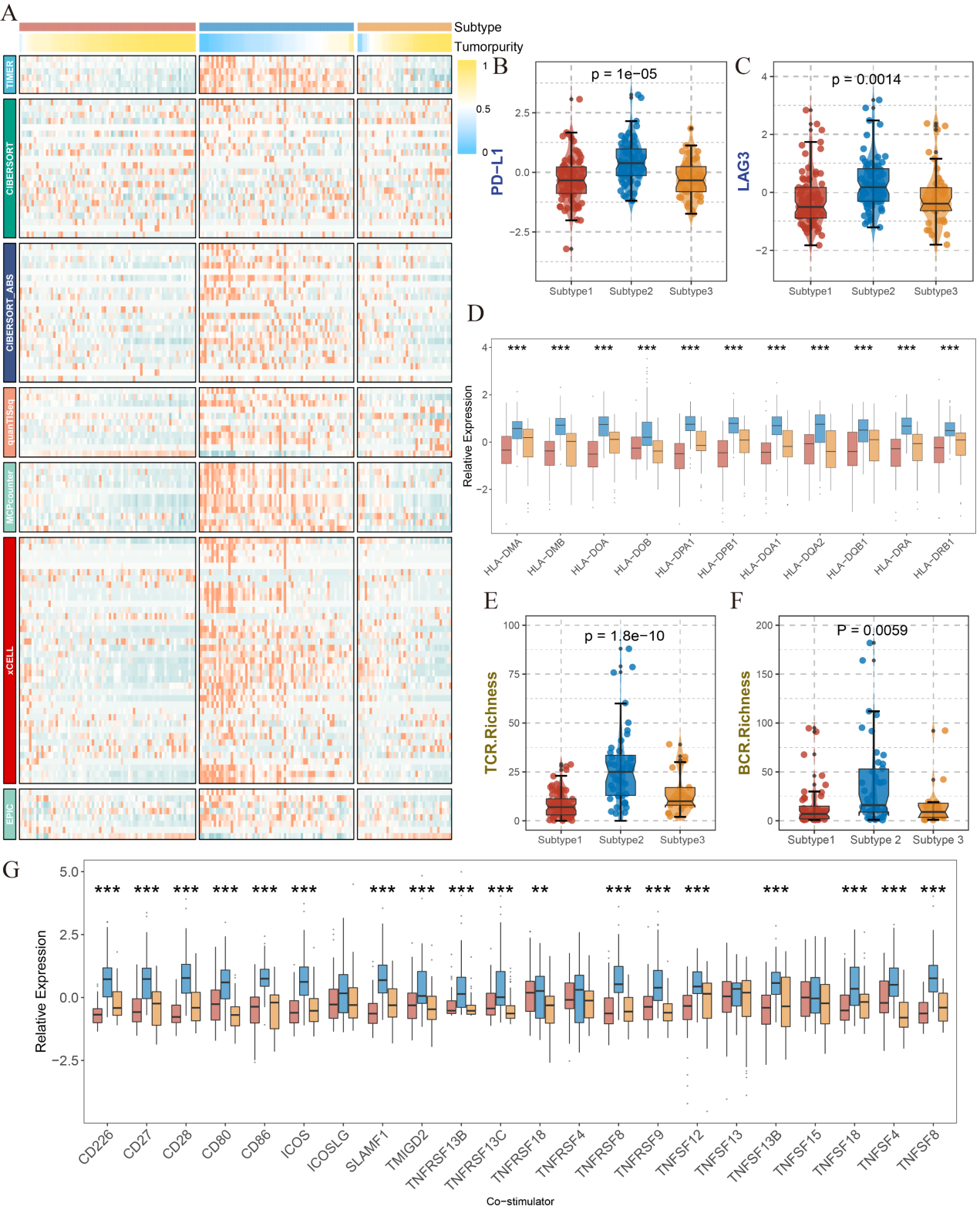
**Supplementary Fig. 2 A.** The heatmap of immune infiltrative abundance of three subtypes by other 7 algorithms, including TIMER, CIBERSORT, CIBERSORT_ABS, quanTIseq, MCPcounter, xCell, and EPIC. **B-D.** PD-L1, LAG3, and MHC molecules distributed among three subtypes, plotted in the form of boxplots. **E-F**. The boxplots elaborated immune indices on three subtypes, inclusive of TCR.Shannon and BCR.Shannon signature score. **G.** The canonical co-inhibitors were pictured across three subtypes.


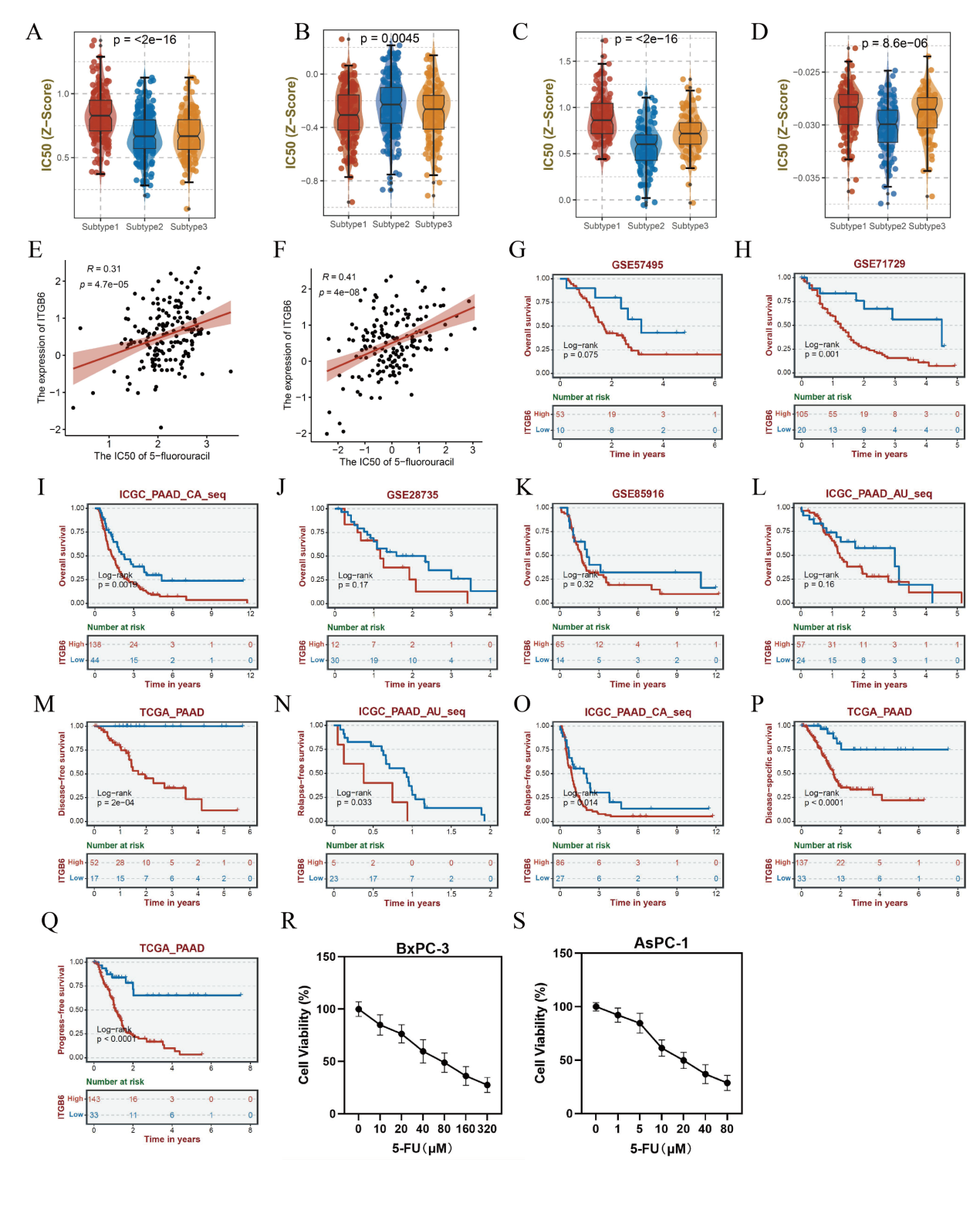
**Supplementary Fig. 3 A-B.** The IC50 value of 5-fluorouracil in PRISM or GDSC across three subtypes in GEO-Meta in the form of boxplot. **C-D.** The IC50 value of 5-fluorouracil in PRISM or GDSC across three subtypes in ICGC-Meta in the form of boxplot. **E-F.** The scatter plot of correlation between ITGB6 expression and IC50 value of 5-fluorouracil in PRISM or GDSC. **G-Q.** Kaplan-Meier (K-M) survival curves for ITGB6 inspected by log-rank test in multiple PAAD datasets. **R-S.** Cell viability at different concentrations of 5-fluorouracil.
